# Test–retest reliability of transfer function analysis metrics for assessing dynamic cerebral autoregulation to spontaneous blood pressure oscillations

**DOI:** 10.1113/EP091500

**Published:** 2024-04-08

**Authors:** Markus Harboe Olsen, Christian Riberholt, Tenna Capion, Ronni R. Plovsing, Kirsten Møller, Ronan M. G. Berg

**Affiliations:** ^1^ Department of Neuroanaesthesiology, The Neuroscience Centre Copenhagen University Hospital – Rigshospitalet Copenhagen Denmark; ^2^ Department of Neurorehabilitation/Traumatic Brain Injury Unit, The Neuroscience Centre Copenhagen University Hospital – Rigshospitalet Copenhagen Denmark; ^3^ Department of Neurosurgery, The Neuroscience Centre Copenhagen University Hospital – Rigshospitalet Copenhagen Denmark; ^4^ Department of Anaesthesia and Intensive Care Copenhagen University Hospital – Hvidovre Copenhagen Denmark; ^5^ Department of Clinical Medicine, Faculty of Health and Medical Sciences University of Copenhagen Copenhagen Denmark; ^6^ Department of Clinical Physiology and Nuclear Medicine, The Diagnostic Centre Copenhagen University Hospital – Rigshospitalet Copenhagen Denmark; ^7^ Centre for Physical Activity Research Copenhagen University Hospital – Rigshospitalet Copenhagen Denmark; ^8^ Department of Biomedical Sciences, Faculty of Health and Medical Sciences University of Copenhagen Copenhagen Denmark; ^9^ Neurovascular Research Laboratory, Faculty of Life Sciences and Education University of South Wales Pontypridd UK

**Keywords:** physiolometrics, sepsis, subarachnoid haemorrhage, transcranial Doppler ultrasound

## Abstract

Transfer function analysis (TFA) is a widely used method for assessing dynamic cerebral autoregulation in humans. In the present study, we assessed the test–retest reliability of established TFA metrics derived from spontaneous blood pressure oscillations and based on 5 min recordings. The TFA‐based gain, phase and coherence in the low‐frequency range (0.07–0.20 Hz) from 19 healthy volunteers, 37 patients with subarachnoid haemorrhage and 19 patients with sepsis were included. Reliability assessments included the smallest real difference (SRD) and the coefficient of variance for comparing consecutive 5 min recordings, temporally separated 5 min recordings and consecutive recordings with a minimal length of 10 min. In healthy volunteers, temporally separating the 5 min recordings led to a 0.38 (0.01–0.79) cm s^−1^ mmHg^−1^ higher SRD for gain (*P *= 0.032), and extending the duration of recordings did not affect the reliability. In subarachnoid haemorrhage, temporal separation led to a 0.85 (−0.13 to 1.93) cm s^−1^ mmHg^−1^ higher SRD (*P* = 0.047) and a 20 (−2 to 41)% higher coefficient of variance (*P *= 0.038) for gain, but neither metric was affected by extending the recording duration. In sepsis, temporal separation increased the SRD for phase by 94 (23–160)° (*P *= 0.006) but was unaffected by extending the recording. A recording duration of 8 min was required to achieve stable gain and normalized gain measures in healthy individuals, and even longer recordings were required in patients. In conclusion, a recording duration of 5 min appears insufficient for obtaining stable and reliable TFA metrics when based on spontaneous blood pressure oscillations, particularly in critically ill patients with subarachnoid haemorrhage and sepsis.

## Abstract (highlights)

- **What is the central question of this study?**
  Current recommendations advise a minimum recording duration of 5 min for assessing dynamic cerebral autoregulation (dCA) by transfer function analysis (TFA). However, the test–retest reliability of spontaneous TFA metrics based on 5 min recordings is unknown, both in healthy individuals and in various patient populations in which dCA might be impaired.
- **What is the main finding and its importance?**
  A 5 min recording duration appears to be insufficient for obtaining stable and reliable TFA‐based dCA when based on spontaneous blood pressure oscillations, particularly in patients with subarachnoid haemorrhage and sepsis.

## 1 INTRODUCTION

Transfer function analysis (TFA), based on continuous measurements of arterial blood pressure (ABP) as the input and transcranial Doppler ultrasound‐based blood velocity in one or more cerebral arteries as the output (Zhang et al., 1998), is undeniably one of the most widely used methods for assessing dynamic cerebral autoregulation (dCA) in humans (Claassen et al., 2021). Despite its sound theoretical background, the generalizability and the subsequent implications for physiological and pathophysiological changes in dCA in various conditions were hampered initially by a substantial lack of standardization of the methodology, including parameter settings, measurement protocols, data analysis and reporting of findings (Meel‐van den Abeelen, Simpson, et al., 2014; Meel‐van den Abeelen, van Beek, et al., 2014). Thus, findings from different centres were incomparable and conclusions could not be generalized, which ultimately hindered progress towards clinical application (Claassen et al., 2016).

In an effort to improve standardization, members of the international Cerebrovascular Research Network (CARNet) published an extensive white paper on the assessment of dCA by TFA (Claassen et al., 2016). Since its original publication, the white paper has led to greater methodological convergence in the field, which, among other things, has facilitated the comparison of outcomes in multicentre studies (Panerai et al., 2023). The white paper was recently updated and currently includes 17 directly applicable in‐depth recommendations on data collection, analysis, interpretation and reporting, based on the best available evidence to date (Panerai et al., 2023). Furthermore, source codes and easy‐to‐use packages for MatLab and R are available free, ensuring replicable results (Olsen et al., 2023).

In evaluating dCA using TFA based on spontaneous ABP oscillations within the low‐frequency range (0.07–0.20 Hz), a minimum recording duration of 5 min is advised to ensure sufficient frequency resolution and stability of TFA metrics (Panerai et al., 2023). Although 5 min recordings have also been deemed sufficient for patients with acute ischaemic stroke (Intharakham et al., 2019), a longer duration of 7 min has been adopted for patients with cerebral artery stenosis (Liu et al., 2021). However, in cases of subarachnoid haemorrhage (SAH) and severe sepsis [conditions in which impaired dCA is a well‐documented and critical aspect of the cerebral pathophysiology (Otite et al., 2014; Taccone et al., 2013)], autonomic nervous system dysfunction, with an altered sympathetic drive to the heart and peripheral vessels, might substantially affect the frequency and magnitude of spontaneous ABP oscillations (Berg et al., 2016; Beseoglu et al., 2010; Yang et al., 2019; Yien et al., 1997). At present, the adequacy of 5 min recordings in these specific conditions remains to be determined.

In the continued efforts to optimize the design of future clinical studies based on TFA‐based metrics and ultimately to promote clinical application, it is imperative to document their test–retest reliability. However, the test–retest reliability of TFA‐based metrics obtained and reported in accordance with the current white paper recommendations from CARNet remains elusive.

In the present study, we formally assessed the test–test reliability of TFA metrics for assessing dCA as based on spontaneous ABP oscillations by a standardized approach (Hartmann et al., 2023), both in healthy individuals and in critically ill patients with SAH and sepsis. We hypothesized that: (1) TFA‐based dCA to spontaneous ABP oscillations based on 5 min recordings would yield inferior test–retest reliability estimates in both patient groups compared with healthy volunteers; (2) the reliability estimates would be more susceptible to temporal separation of recordings in patients than in healthy volunteers; and (3) extending the recording duration would improve reliability in all groups.

## 2 MATERIALS AND METHODS

### 2.1 Ethical approval

The present retrospective work is based on data from four studies, which have previously been published elsewhere (Berg et al., 2015, 2012; Berg, Plovsing, et al., 2013; Berg & Plovsing, 2016; Olsen et al., 2022), and describes entirely separate analyses to address an independent working hypothesis. All studies were approved by either the Scientific Ethical Committee of Copenhagen and Frederiksberg Municipalities or the Capital Region of Copenhagen (file numbers H‐A‐2009‐020, H‐2‐2010‐04 and H‐19017185) and conformed to the standards set by the most recent version of the *Declaration of Helsinki* (WMA, 2013), except for registration in a database. The healthy subjects provided oral and written informed consent prior to inclusion. The patients were all temporarily incapacitated at inclusion owing to unconsciousness, and in accordance with Danish legislation and the approvals from the Scientific Ethical Committee, oral and written proxy informed consent was obtained from the next‐of‐kin (Berg, Møller, et al., 2013). When patients regained consciousness, oral and written informed consent was also obtained from them.

### 2.2 Subjects

The four original studies recorded invasive ABP in the radial artery and transcranial Doppler ultrasound‐based middle cerebral artery blood velocity (MCAv) in a total of 19 healthy volunteers (with 19 individual recordings) (Berg et al., 2015, 2012; Berg, Plovsing, et al., 2013), in 37 patients with SAH (with 59 individual recordings) (Olsen et al., 2022) and in 19 patients admitted to the intensive care unit with severe sepsis (with 35 individual recordings) (Berg & Plovsing, 2016; Berg et al., 2012). All subjects were placed in the supine position, with slight head elevation (20°), and all included recordings were obtained after 20 min of rest after instrumentation and before any study intervention was carried out, and all are ≥10 min long, but varied between participants and studies, and were ≤38 min long. The experimental set‐ups are described in full in the original publications (Berg et al., 2015, 2012; Berg, Plovsing, et al., 2013; Berg & Plovsing, 2016; Olsen et al., 2022).

### 2.3 Data processing

The recordings were extracted from LabChart into a tab‐delimited file at the original resolution of 1000 Hz and visually inspected for artefacts. Any artefacts were deleted by removing the associated period that started and ended in a curve nadir. The TFA function from the publicly available R package ‘clintools’ was used to calculate the TFA‐based metrics, normalized gain, non‐normalized gain and phase in the low‐frequency range (0.07–0.20 Hz) (Olsen et al., 2023; Panerai et al., 2023). Processing was conducted in accordance with recommendations of the recent CARNet white paper (Panerai et al., 2023). This involved interpolation of artefacts in the raw recordings if no more than three consecutive beats were missing, which were then passed through a Hanning window with a duration of 102.4 s and a maximum of 59.99% overlap, after which fast discrete Fourier transformation was applied. The maximum overlap of 59.99% was set to allow for the maximum coverage of the data according to the default by the two publicly available packages (Olsen et al., 2023). Furthermore, a coherence threshold was applied using 95% confidence limits based on degrees of freedom, and all frequencies with low‐magnitude‐squared coherence were excluded from averaging when calculating the mean values. For details, please also see Olsen et al. (2023).

### 2.4 Point of stability

To assess the required length of a time series to provide a stable TFA metric, we used the expanding window sensitivity method (Mahdi et al., 2017; Schönbrodt & Perugini, 2013). Here, the expanding window sensitivity (*E*) of a given TFA metric is calculated for each increment of the time series, which represents the average variability in this metric over time. Defining a corridor of stability [here defined as the 95% confidence interval around the expanding window sensitivity at a recording length of 15 min (three times the recommended 5 min recording length)] allows for identification of the point of stability. The point of stability is defined as the recording duration at which the mean expanding window sensitivity no longer leaves the corridor of stability. Hence, the point of stability is the minimal data recording length required for a TFA metric to remain stable. This has been suggested as the minimal recording length necessary to ensure a valid TFA metric (Mahdi et al., 2017).

### 2.5 Statistics

All statistical analyses were carried out using R v.4.2.1 (R Core Team, Vienna, Austria). If not specified, normally distributed data are presented as the mean (±SD) and non‐normally distributed data as the median [interquartile range (IQR)]. The data analysis was based on the following: (1) a comparison of consecutive 5 min recordings; (2) a comparison of the first versus last 5 min of each recording; (3) a comparison of the first versus last half of all recordings; (4) identifying the minimal recommended recording duration using the above‐described point of stabilization methodology for each of the TFA‐based metrics; and (5) a comparison of the first versus last half of all recordings with a recording duration at least twice the identified minimal recommended recording duration.

Reliability was evaluated in terms of both absolute and relative reliability, both of which refer to either the repeatability or the reproducibility of a measurement, of which the former refers to the ability of a method to obtain the same results in identical conditions, whereas the latter reflects the ability to obtain the same results in changing conditions (Bartlett & Frost, 2008). The reliability estimates obtained in the present study are within the repeatability domain.

Student's paired *t*‐test was applied to ensure internal consistency (i.e., the absence of systematic error between recording segments at the group level); for variables that were not internally consistent, no reliability estimates were reported. For the assessment of absolute and relative reliability, the publicly available *calcrel* function in the *clintools* package in R was used (Olsen et al., 2023). For absolute reliability, this involved Bland–Altman analysis‐based limits of agreement (LOA) and the closely related smallest real difference (SRD), which estimates the maximum difference between any two measurements on 95% of the occasions, using a one‐way ANOVA (Vaz et al., 2013). Relative reliability was assessed by the coefficient of variation (CV), and, based on the distribution of the estimates of mean and residual variance from a linear mixed model, the distribution of the 95% confidence intervals was obtained (Liu, 2012). Given that phase included both positive and negative values, it was not mathematically possible to derive CV. Furthermore, the two‐way mixed‐effects single measurement agreement intraclass correlation coefficient (ICC) was also used as a measure of relative reliability. Differences between reliability measures were assessed by bootstrapping using the *comparerel* function from the *clintools* package (Olsen et al., 2023). Through 1000 iterations, the difference between the measures was calculated, together with the confidence intervals for the difference and the *P*‐value.

## 3 RESULTS

Baseline characteristics of study participants and recordings are provided in Table 1. All recordings showed internal consistency for all reliability estimates, except for phase in healthy volunteers when comparing the first half with the last half of the recordings, and coherence in SAH when comparing consecutive 5 min recordings (data not shown).

**TABLE 1:** Patient and recording characteristics.

Characteristic	Healthy (*n* = 19)	SAH (*n* = 37)	Sepsis (*n* = 19)
Age, years, median (IQR)	23 (21.5–24.5)	57 (51–64)	64 (50–70)
Male, *n* (%)	19 (100)	7 (19)	17 (89)
Mortality, *n* (%)	0 (0)	6 (16)	5 (26)
Recordings, *n*	19	59	35
Recording duration, min, median (IQR)	19.2 (16.5–19.9)	24.9 (17.4–29)	15 (14.7–16.7)
Recordings longer than 24 min, *n*	1	34	1
Recordings longer than 15 min, *n*	17	50	17
$P_{\mathrm{aC}O_{2}}$, kPa, mean + SD	5.6 + 0.3	5.1 + 0.5	5.6 + 1.4
ABP, mmHg, mean + SD	90 + 5.9	82.5 + 35.8	76.2 + 9.9
MCAv, cm s^−1^, mean + SD	69.6 + 11.7	67.7 + 24.4	64.7 + 42.3

Abbreviations: ABP, arterial blood pressure; MCAv, middle cerebral artery blood flow velocity; SAH, subarachnoid haemorrhage.

### 3.1 Healthy volunteers

All reliability estimates for consecutive 5 min recordings, temporally separated 5 min recordings, and for the first half versus the last half of the recording are provided in Table 2. In comparison to consecutive 5 min recordings, temporal separation led to a higher SRD for gain and a wider LOA for phase, with a similar trend for normalized gain and coherence (Table 3). Extending the recordings to the first half versus last half did not affect the reliability estimates for any TFA metric.

**TABLE 2:** Test–retest reliability of transfer function analysis metrics for assessing dynamic cerebral autoregulation in humans.

Group	Variable	*n*	Segment duration (min)	Time between segments (min)	Absolute reliability		Relative reliability	
					Bias (units)	SRD (units)	CV (%)	ICC (fraction)
Healthy	Gain, cm s^−1^ mmHg^−1^	18	5	0	0 (−0.3 to 0.3)	0.29 (0.23–0.37)	7.78 (5.3–10.3)	0.71 (0.37–0.88)
		19	5	8.5 (7.7–9.3)	0 (−0.7 to 0.6)	0.66 (0.38–1.57)	18.99 (12.9–25.1)	0.11 (−0.38 to 0.54)
		19	9.2 (8.4–10.1)	0	0 (−0.3 to 0.3)	0.29 (0.22–0.41)	8.76 (6–11.6)	0.9 (0.76–0.96)
	N‐gain, % mmHg^−1^	18	5	0	0 (−0.5 to 0.5)	0.51 (0.41–0.65)	9.49 (6.4–12.6)	0.71 (0.36–0.88)
		19	5	8.5 (7.7–9.3)	0 (−1 to 1)	1.04 (0.62–2.26)	20.42 (13.9–27.1)	−0.02 (−0.49 to 0.44)
		19	9.2 (8.4–10.1)	0	0 (−0.5 to 0.4)	0.48 (0.35–0.71)	9.92 (6.8–13.1)	0.88 (0.71–0.95)
	Phase, degrees	18	5	0	2.8 (−23.1 to 28.8)	27.81 (22.04–36.62)	–	0.63 (0.26–0.84)
		19	5	8.5 (7.7–9.3)	17.9 (−57.3 to 93.2)	87.1 (44.37–291.59)	–	0.39 (−0.02 to 0.7)
		19	9.2 (8.4–10.1)	0	–	–	–	–
	Coherence, fraction	18	5	0	0 (−0.2 to 0.1)	0.19 (0.15–0.25)	10.31 (7–13.7)	0.79 (0.53–0.92)
		19	5	8.5 (7.7–9.3)	0 (−0.3 to 0.4)	0.37 (0.22–0.82)	20.42 (13.9–27.1)	0.36 (−0.11 to 0.69)
		19	9.2 (8.4–10.1)	0	0 (−0.1 to 0.2)	0.18 (0.15–0.22)	10.59 (7.2–13.9)	0.9 (0.75–0.96)
SAH	Gain, cm s^−1^ mmHg^−1^	32	5	0	−0.1 (−1.4 to 1.2)	1.31 (0.83–2.53)	32.55 (24.2–41.5)	0.91 (0.82–0.95)
		38	5	13.6 (12.5–14.6)	−0.1 (−2.2 to 2)	2.16 (1.57–3.25)	52.74 (39.7–67.1)	0.61 (0.37–0.78)
		38	11.7 (10.8–12.6)	0	0 (−0.5 to 0.6)	0.57 (0.42–0.84)	36.58 (27.9–45.8)	0.83 (0.69–0.91)
	N‐gain, % mmHg^−1^	32	5	0	−0.1 (−2 to 1.7)	1.9 (1.3–3.18)	29 (21.6–36.9)	0.92 (0.85–0.96)
		38	5	13.6 (12.5–14.6)	−0.2 (−3.4 to 3)	3.28 (2.38–4.95)	50.5 (38.2–64.2)	0.67 (0.46–0.82)
		38	11.7 (10.8–12.6)	0	0 (−0.6 to 0.7)	0.65 (0.48–0.94)	42.24 (32.1–53.2)	0.83 (0.7–0.91)
	Phase, degrees	28	5	0	–	–	–	–
		36	5	13.6 (12.5–14.6)	−0.1 (−96.3 to 96)	98.23 (70.58–150.09)	–	0.48 (0.17–0.69)
		35	11.7 (10.8–12.6)	0	−0.4 (−66.4 to 65.6)	67.47 (51.04–95.04)	–	0.72 (0.51–0.85)
	Coherence, fraction	58	5	0	−0.1 (−0.4 to 0.3)	0.37 (0.29–0.5)	40.18 (32.4–48.3)	0.63 (0.43–0.77)
		58	5	13.6 (12.5–14.6)	0 (−0.5–0.4)	0.44 (0.35–0.58)	45.78 (36.8–55.3)	0.5 (0.28–0.67)
		58	11.7 (10.8–12.6)	0	0 (−0.4 to 0.4)	0.39 (0.31–0.53)	51.09 (41–62.1)	0.61 (0.42–0.75)
Sepsis	Gain, cm s^−1^ mmHg^−1^	18	5	0	0.1 (−1.3 to 1.4)	1.42 (1.05–2.05)	28.36 (18.9–38.5)	0.81 (0.55–0.92)
		22	5	8.1 (7.6–8.6)	0.2 (−1.4 to 1.8)	1.72 (1.12–3.16)	33.17 (23.1–44)	0.75 (0.49–0.89)
		20	8.1 (7.6–8.6)	0	0 (−1.1 to 1.1)	1.15 (0.78–1.94)	27.02 (18.4–36)	0.8 (0.57–0.92)
	N‐gain, % mmHg^−1^	18	5	0	0.2 (−3 to 3.4)	3.34 (2.2–5.98)	40.05 (26.4–55.5)	0.73 (0.41–0.89)
		22	5	8.1 (7.6–8.6)	0.4 (−3.2 to 3.9)	3.77 (2.46–6.86)	42.15 (29.1–56.8)	0.68 (0.38–0.85)
		20	8.1 (7.6–8.6)	0	−0.2 (−2.1 to 1.8)	2.06 (1.43–3.32)	28.68 (19.6–38.4)	0.79 (0.55–0.91)
	Phase, degrees	16	5	0	4.2 (−49.6 to 58)	57.37 (38.74–97.58)	–	0.62 (0.2–0.85)
		21	5	8.1 (7.6–8.6)	−21.9 (−160.8 to 116.9)	151.31 (103.52–251.35)	–	0.24 (−0.18 to 0.59)
		19	8.1 (7.6–8.6)	0	8.8 (−77.3 to 94.8)	91.66 (59.31–169.04)	–	0.16 (−0.31 to 0.57)
	Coherence, fraction	35	5	0	0 (−0.2 to 0.3)	0.25 (0.2–0.33)	32.31 (24.4–40.6)	0.81 (0.66–0.9)
		35	5	8.1 (7.6–8.6)	0 (−0.3 to 0.3)	0.3 (0.23–0.43)	40.36 (30.3–51)	0.71 (0.49–0.84)
		35	8.1 (7.6–8.6)	0	0 (−0.3 to 0.3)	0.32 (0.23–0.49)	51.2 (38–65.7)	0.71 (0.49–0.84)

Abbreviations: CV, coefficient of variation; ICC, intraclass correlation coefficient; N‐gain, normalized gain; SAH, subarachnoid haemorrhage; SRD, smallest real difference.

**TABLE 3:** Within‐group differences in test–retest reliability measures of transfer function analysis metrics in comparison to consecutive 5 min recordings.

	*n*	Absolute reliability				Relative reliability			
		Bias units (95% LOA)	*P*‐value	SRD units (95% CI)	*P*‐value	CV % (95% CI)	*P*‐value	ICC fraction (95% CI)	*P*‐value
**Healthy**									
**Temporally separated 5 min recordings**									
Gain, cm s^−1^ mmHg^−1^	19	0 (−0.2; 0.2)	0.663	**−0.38 (−0.79; 0.01)**	**0.032**	**−11.22 (−23.78; 0)**	**0.025**	0.6 (−0.27; 1.18)	0.129
N‐gain, % mmHg^−1^	19	0 (−0.2; 0.3)	0.557	−0.53 (−1.16; 0.06)	0.062	**−10.92 (−23.99; 0.8)**	**0.044**	0.72 (−0.1; 1.3)	0.051
Phase, degrees	19	**−15.1 (−33.66; −1)**	**0.017**	−59.29 (−119.71; 5.39)	0.134	–	–	0.25 (−0.4; 0.47)	0.303
Coherence, fraction	19	0 (−0.2; 0)	0.564	−0.19 (−0.4; 0.03)	0.060	−10.11 (−23.29; 2)	0.081	0.43 (−0.1; 0.89)	0.079
**First versus last half of recordings**									
Gain, cm s^−1^ mmHg^−1^	19	0 (−0.1; 0.1)	0.889	−0.01 (−0.11; 0.11)	0.459	−0.98 (−4.37; 2.68)	0.299	−0.19 (−0.6; 0.16)	0.145
N‐gain, % mmHg^−1^	19	0 (−0.1; 0.2)	0.607	0.03 (−0.16; 0.23)	0.375	−0.43 (−4.43; 3.54)	0.457	−0.17 (−0.58; 0.2)	0.145
Phase, derees	19	–	–	–	–	–	–	–	–
Coherence, fraction	19	0 (−0.1; 0)	0.760	0.01 (−0.05; 0.07)	0.388	−0.27 (−4.68; 3.67)	0.418	−0.1 (−0.36; 0.11)	0.168
**SAH**									
**Temporally separated 5 min recordings**									
Gain, cm s^−1^ mmHg^−1^	38	0 (−0.4; 0.4)	0.532	**−0.85 (−1.93; 0.13)**	**0.047**	**−20.19 (−40.96; 1.97)**	**0.038**	0.3 (−0.09; 0.67)	0.057
N‐gain, % mmHg^−1^	38	0.1 (−0.6; 0.7)	0.479	**−1.38 (−2.85; 0.16)**	**0.036**	**−21.5 (−39.58; 0.12)**	**0.027**	0.25 (−0.09; 0.56)	0.060
Phase, degrees	36	0.3 (−21.59; 21.7)	0.479	−17.6 (−70.82; 34.21)	0.263	–	–	0.12 (−0.9; 0.83)	0.398
Coherence, fraction	58	–	–	–	–	–	–	–	–
**First versus last half of recordings**									
Gain, cm s^−1^ mmHg^−1^	38	−0.1 (−0.4; 0.1)	0.238	**0.74 (0.06; 1.38)**	**0.016**	−4.03 (−25.52; 15.17)	0.308	0.08 (−0.3; 0.27)	0.281
N‐gain, % mmHg^−1^	38	−0.1 (−0.5; 0.2)	0.275	**1.25 (0.43; 2.07)**	**<0.001**	−13.23 (−35.2; 6.61)	0.091	0.09 (−0.22; 0.34)	0.202
Phase, degrees	35	0.6 (−18.42; 18.55)	0.479	13.16 (−31.33; 50.56)	0.278	–	–	−0.12 (−1.09; 0.35)	0.318
Coherence, fraction	58	–	–	–	–	–	–	–	–
**Sepsis**									
**Temporally separated 5 min recordings**									
Gain, cm s^−1^ mmHg^−1^	22	−0.1 (−0.7; 0.24)	0.298	−0.3 (−1.29; 0.66)	0.295	−4.82 (−21.79; 12.44)	0.348	0.06 (−0.25; 0.42)	0.395
N‐gain, % mmHg^−1^	22	−0.2 (−1.2; 0.9)	0.406	−0.43 (−2.94; 2.08)	0.400	−2.1 (−22.2; 20.11)	0.462	0.05 (−0.22; 0.27)	0.369
Phase, degrees	21	26.1 (−5.28; 61.39)	0.067	**−93.94 (−160.08; −22.7)**	**0.006**	**–**	**–**	0.39 (−0.31; 0.99)	0.127
Coherence, fraction	35	0 (0; 0.1)	0.945	−0.05 (−0.17; 0.06)	0.196	−8.05 (−23.31; 7.51)	0.160	0.11 (−0.16; 0.4)	0.182
**First versus last half of recordings**									
Gain, cm s^−1^ mmHg^−1^	20	0.1 (−0.3; 0.5)	0.406	0.27 (−0.44; 0.92)	0.198	1.34 (−12.24; 15.61)	0.381	0 (−0.29; 0.32)	0.475
N‐gain, % mmHg^−1^	20	0.4 (−0.4; 1.2)	0.224	1.28 (−0.64; 3.06)	0.101	11.38 (−6.22; 24.23)	0.101	−0.07 (−0.25; 0.17)	0.234
Phase, degrees	19	−4.6 (−27.69; 20)	0.376	−34.29 (−82.25; 18.89)	0.127	**–**	**–**	0.46 (−0.31; 0.91)	0.122
Coherence, fraction	35	0 (−0.1; 0.1)	0.958	−0.07 (−0.21; 0.06)	0.152	**−18.89 (−39.12; 2.6)**	**0.042**	0.1 (−0.17; 0.43)	0.220

Abbreviations: CV, coefficient of variation; ICC, intraclass correlation coefficient; N‐gain, normalized gain; SAH, subarachnoid haemorrhage; SRD, smallest real difference.

### 3.2 Subarachnoid haemorrhage

All reliability estimates for consecutive 5 min recordings, temporally separated 5 min recordings, and for the first half versus the last half of the recordings are provided in Table 2. In comparison to consecutive 5 min recordings, temporal separation led to higher SRD and CV for gain and normalized gain, and all reliability estimates were unaffected for the remaining TFA metrics (Table 3). Extending the recordings to the first half versus last half decreased the SRD for gain and normalized gain (Table 3).

Comparisons of the absolute and relative reliability estimates of all TFA metrics of SAH patients with those of healthy volunteers as based on consecutive 5 min recordings, temporally separated 5 min recordings, and the first half versus last half of recordings are provided in Table 4.

**TABLE 4:** Difference in test–retest reliability of transfer function analysis metrics compared with healthy volunteers.

	*n*	Absolute reliability				Relative reliability			
		Bias units (95% LOA)	*P*‐value	SRD units (95% CI)	*P*‐value	CV % (95% CI)	*P*‐value	ICC fraction (95% CI)	*P*‐value
**SAH**									
**Consecutive 5 min recordings**									
Gain, cm s^−1^ mmHg^−1^	32	0.1 (−0.1; 0.3)	0.385	−1.03 (−1.69; −0.36)	**0.000**	**−24.77 (−41.88; −9.86)**	**0.000**	−0.19 (−0.6; 0.19)	0.128
N‐gain, % mmHg^−1^	32	0.1 (−0.2; 0.5)	0.317	−1.4 (−2.24; −0.57)	**0.000**	**−19.51 (−34.9; −7.61)**	**0.000**	−0.22 (−0.65; 0.09)	0.064
Phase, degrees	28	2.6 (−13.5; 18.5)	0.368	−52.82 (−85.19; −14.4)	**0.005**	–	–	0.04 (−0.47; 0.95)	0.436
Coherence, fraction	58	–	–	–	–	–	–	–	–
**Temporally separated 5 min recordings**									
Gain, cm s^−1^ mmHg^−1^	38	0.1 (−0.3; 0.5)	0.429	**−1.5 (−2.36; −0.59)**	**0.000**	**−33.75 (−50.11; −14.8)**	**0.002**	−0.5 (−1.06; 0.28)	0.180
N‐gain, % mmHg^−1^	38	0.2 (−0.4; 0.7)	0.347	**−2.25 (−3.45; −0.64)**	**0.003**	**−30.08 (−47.82; −7.51)**	**0.003**	−0.69 (−1.24; 0.1)	0.053
Phase, degrees	36	18 (−4.6; 42.54)	0.060	−11.13 (−97.47; 67.56)	0.321	–	–	−0.09 (−0.42; 0.71)	0.438
Coherence, fraction	58	0 (0; 0.2)	0.447	−0.07 (−0.31; 0.16)	0.239	**−25.35 (−42.83; −9.24)**	**0.003**	−0.14 (−0.64; 0.41)	0.387
**First versus last half of recordings**									
Gain, cm s^−1^ mmHg^−1^	38	0 (−0.1; 0.1)	0.532	**−0.32 (−0.52; −0.13)**	**0.000**	−4.27 (−19.43; 11.74)	0.279	−0.02 (−0.22; 0.17)	0.446
N‐gain, % mmHg^−1^	38	0 (−0.1; 0.1)	0.683	**−0.4 (−0.61; −0.15)**	**0.000**	−9.93 (−29.91; 7.86)	0.158	−0.02 (−0.25; 0.23)	0.428
Phase, degrees	35	–	–	–	–	–	–	–	–
Coherence, fraction	58	0 (0; 0.1)	0.755	**−0.14 (−0.27; −0.01)**	**0.017**	**−18.78 (−36.78; −1.34)**	**0.015**	0.2 (−0.07; 0.48)	0.071
**Sepsis**									
**Consecutive 5 min recordings**									
Gain, cm s^−1^ mmHg^−1^	18	−0.1 (−0.4; 0.3)	0.488	**−1.13 (−1.56; −0.64)**	**0.000**	**−20.58 (−28.9; −13.04)**	**0.000**	−0.1 (−0.52; 0.19)	0.262
N‐gain, % mmHg^−1^	18	−0.2 (−0.9; 0.6)	0.357	**−2.83 (−4.32; −1.03)**	**0.000**	**−30.56 (−38.89; −12.92)**	**0.000**	−0.02 (−0.46; 0.19)	0.386
Phase, degrees	16	−1.4 (−16.7; 11.1)	0.429	**−29.55 (−54.7; −1.25)**	**0.021**	**–**	–	0.01 (−0.5; 0.62)	0.498
Coherence, fraction	35	0 (−0.1; 0)	0.775	−0.06 (−0.14; 0.02)	0.070	**−22 (−33.16; −12.73)**	**0.000**	−0.02 (−0.31; 0.2)	0.410
**Temporally separated 5 min recordings**									
Gain, cm s^−1^ mmHg^−1^	22	−0.2 (−0.6; 0.1)	0.173	**−1.06 (−1.99; −0.12)**	**0.014**	−14.18 (−33.79; 6)	0.112	−0.63 (−1.24; 0.1)	0.061
N‐gain, % mmHg^−1^	22	−0.4 (−1.2; 0.4)	0.195	**−2.73 (−4.57; −0.5)**	**0.002**	**−21.74 (−39.17; 1.44)**	**0.036**	**−0.7 (−1.26; 0.01)**	**0.030**
Phase, degrees	21	**39.8 (7.32; 77.06)**	**0.008**	−64.21 (−166.58; 30.14)	0.107	–	–	0.15 (−0.29; 0.91)	0.236
Coherence, fraction	35	0 (0; 0.1)	0.715	0.07 (−0.16; 0.32)	0.364	**−19.94 (−37.46; −1.08)**	**0.013**	−0.35 (−0.86; 0.22)	0.192
**First versus last half of recordings**									
Gain, cm s^−1^ mmHg^−1^	20	0 (−0.2; 0.3)	0.559	**−0.9 (−1.37; −0.34)**	**0.000**	5.29 (−9.09; 22.82)	0.217	0.01 (−0.25; 0.33)	0.492
N‐gain, % mmHg^−1^	20	0.2 (−0.3; 0.6)	0.337	**−1.81 (−2.63; −0.85)**	**0.000**	3.63 (−10.25; 18.07)	0.300	0.02 (−0.19; 0.27)	0.443
Phase, degrees	19	–	–	–	–	–	–	–	–
Coherence, fraction	35	0 (−0.1; 0.1)	0.958	−0.07 (−0.21; 0.06)	0.152	**−18.89 (−39.12; 2.6)**	**0.042**	0.1 (−0.17; 0.43)	0.220

Abbreviations: CV, coefficient of variation; ICC, intraclass correlation coefficient; N‐gain, normalized gain; SAH, subarachnoid haemorrhage; SRD, smallest real difference.

### 3.3 Sepsis

The reliability estimates for consecutive 5 min recordings, temporally separated 5 min recordings, and the first half versus the last half of the recordings are shown in Table 2. In comparison to consecutive 5 min recordings, temporal separation increased the SRD for phase but did not affect any other reliability estimate of any of the other TFA metrics. In the first half versus last half of recordings, CV became higher for coherence, but no other reliability estimate was affected for any other TFA metric.

Comparisons of the absolute and relative reliability estimates of all TFA metrics of sepsis patients with those of healthy volunteers as based on consecutive 5 min recordings, temporally separated 5 min recordings, and the first half versus last half of recordings are provided in Table 4.

### 3.4 Point of stability

**FIGURE 1:**
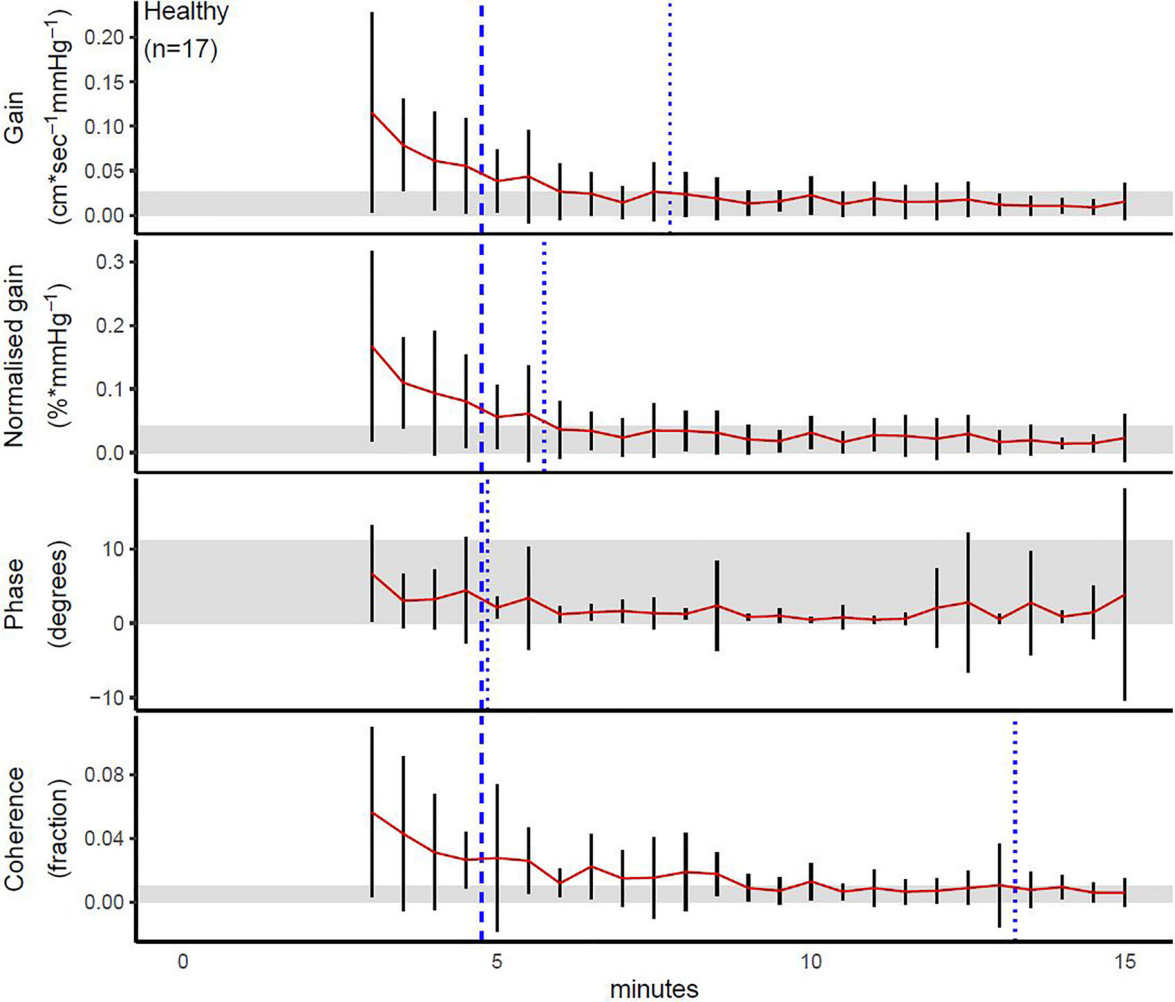
Point of stability for transfer function analysis metrics in healthy volunteers. The bold dashed line shows the recommended recording duration according to the current CARNet recommendations (Panerai et al., 2023), whereas the thin dashed line shows that defined by the point of stability in the present study; these are placed immediately before the data point of interest in order not to obscure the variance estimates. The red line indicates the point estimate as data accumulate over time, and the black vertical lines depict its 95% confidence interval at a given time. The shaded area shows the corridor of stability.

**FIGURE 2:**
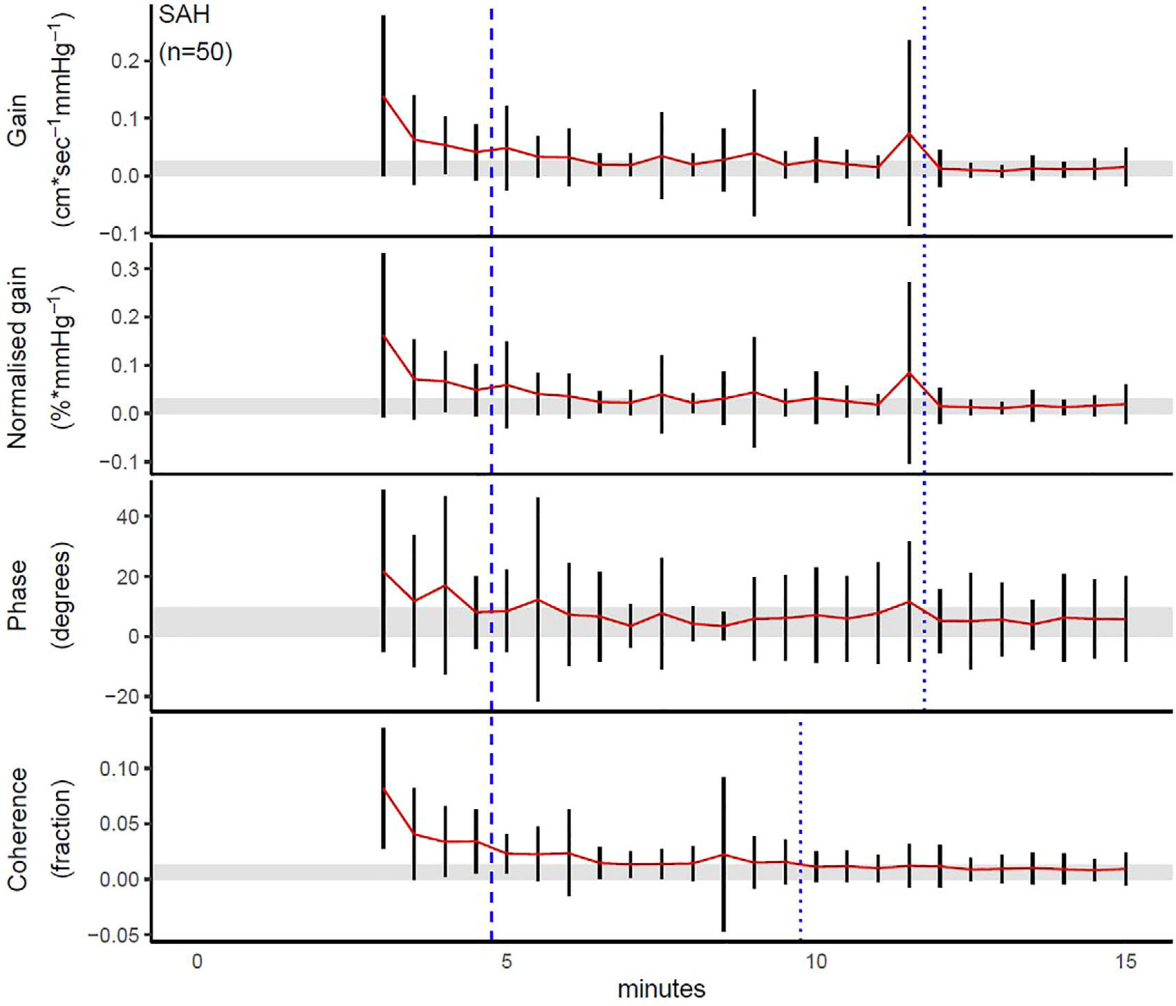
Point of stability for transfer function analysis metrics in patients with subarachnoid haemorrhage. The bold dashed line shows the recommended recording duration according to the current CARNet recommendations (Panerai et al., 2023), whereas the thin dashed line shows that defined by the point of stability in the present study; these are placed immediately before the data point of interest in order not to obscure the variance estimates. The red line indicates the point estimate as data accumulate over time, and the black vertical lines depict its 95% confidence interval at a given time. The shaded area shows the corridor of stability.

**FIGURE 3:**
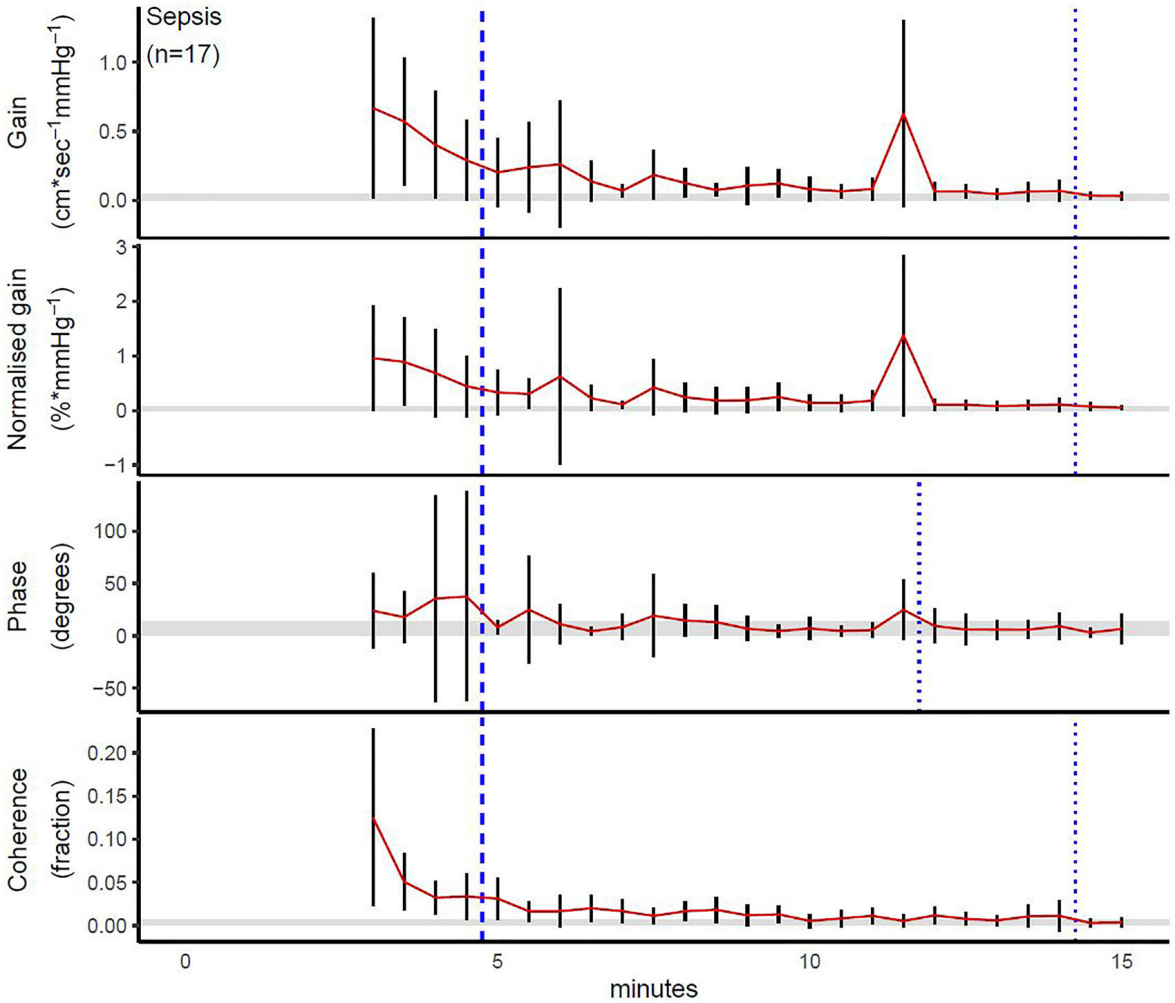
Point of stability for transfer function analysis metrics in patients with sepsis. The bold dashed line shows the recommended recording duration according to the current CARNet recommendations (Panerai et al., 2023), whereas the thin dashed line shows that defined by the point of stability in the present study; these are placed immediately before the data point of interest in order not to obscure the variance estimates. The red line indicates the point estimate as data accumulate over time, and the black vertical lines depict its 95% confidence interval at a given time. The shaded area shows the corridor of stability.

For healthy volunteers, the point of stability was 8 min for gain, 6 min for normalized gain and 13.5 min for coherence; phase never left the corridor of stability (Figure 1). For patients with SAH, gain, normalized gain and phase all stabilized after 12 min, and coherence stabilized after 10 min (Figure 2). For patients with sepsis, gain, normalized gain and coherence all stabilized after 14.5 min, and the point of stability was reached after 12 min for phase (Figure 3). Test–retest reliability data from all the SAH patents who had a minimum recording length of 24 min are provided in Table 5, in which the reliability estimates of the first and next 12 min are provided and compared with consecutive 5 min recordings; apart from a higher ICC for coherence, all absolute and relative reliability estimates were similar for all TFA metrics.

**TABLE 5:** Test–retest reliability of transfer function analysis metrics for assessing dynamic cerebral autoregulation based on consecutive 12 min recordings in subarachnoid haemorrhage.

Variable	*n*	Recording duration (min)	Time between recordings (min)	Absolute reliability				Relative reliability			
				Bias units (95% LOA)	*P*‐value	SRD units (95% CI)	*P*‐value	CV % (95% CI)	*P*‐value	ICC fraction (95% CI)	*P*‐value
Gain, cm s^−1^ mmHg^−1^	34	12	0	−0.1 (−0.8; 0.7)	–	0.8 (0.61; 1.14)	–	28.73 (21.7; 36.1)	–	0.8 (0.65; 0.9)	–
Difference from consecutive 5 min recordings				0 (−0.3; 0.2)	0.541	0.51 (−0.19; 1.23)	0.120	3.82 (−14.74; 24.04)	0.440	0.1 (−0.32; 0.35)	0.236
N‐gain, % mmHg^−1^	34	12	0	−0.1 (−1.3; 1.2)		1.31 (0.97; 1.92)		30.55 (23; 38.3)		0.79 (0.62; 0.89)	–
Difference from consecutive 5 min recordings				0 (−0.4; 0.3)	0.497	0.59 (−0.34; 1.53)	0.115	−1.54 (−17.88; 16.33)	0.400	0.13 (−0.23; 0.54)	0.147
Phase, degrees	34	12	0	−2.2 (−79.1; 74.6)	–	78.7 (63.6; 100.88)		–	–	0.48 (0.17; 0.7)	–
Difference from consecutive 5 min recordings				2.4 (−15.93; 23.49)	0.402	1.93 (−44.36; 39.33)	0.456	–	–	0.11 (−0.88; 0.53)	0.419
Coherence, fraction	34	12	0	0 (−0.3; 0.3)	–	0.29 (0.22; 0.43)	–	34.53 (26; 43.6)	–	0.82 (0.67; 0.91)	–
Difference from consecutive 5 min recordings				−0.1 (−0.1; 0)	0.319	0.08 (−0.05; 0.22)	0.115	5.64 (−12.84; 23.25)	0.272	−0.19 (−0.4; 0.03)	0.049

Abbreviations: CV, coefficient of variation; ICC, intraclass correlation coefficient; N‐gain, normalized gain; SAH, subarachnoid haemorrhage; SRD, smallest real difference.

## 4 DISCUSSION

In this study, we examined the test–retest reliability of TFA‐based assessments of dCA to spontaneous ABP oscillations in human subjects. We found that, in contrast to healthy volunteers, temporal separation of 5 min recordings of spontaneous ABP oscillations and MCAv markedly reduced both absolute and relative reliability estimates for key TFA metrics in both groups, and extending the duration of consecutive recordings did not have any consistent effect on the reliability of any TFA metric in any of the groups.

The absolute reliability focuses on the magnitude or extent of measurement error (quantified in the same unit as the measure of interest), whereas the relative reliability provides information about the relative contribution of measurement error to the overall variation in the measure (provided as a fraction or a percentage) (Hartmann et al., 2023). For example, as an absolute reliability measure, the SRD estimates how much two consecutively obtained measurements in identical conditions will differ in 95% of the occasions, whereas the CV permits the comparison of different metrics, both in the same and in different conditions. It is difficult to define a threshold for CV below which relative reliability might be classified as ‘acceptable’, but it is notable that gain and normalized gain based on consecutive 5 min recordings in healthy volunteers were the only two estimates that did not exceed a CV of 20%, a threshold that has previously been used for test–retest reliability assessments of TFA (Smirl et al., 2015). Gain and normalized gain in healthy volunteers thus also provided the lowest SRD values across the three groups, but for physiological interpretation, the reported SRD of 0.29 cm s^−1^ mmHg^−1^ for gain and 0.51% mmHg^−1^ for normalized gain based on consecutive 5 min recordings in healthy values are nevertheless exceedingly high, because both largely resemble previously reported group‐level changes evoked by moderate hypo‐ and hypercapnia (Tzeng et al., 2012; Zhang et al., 1998). Hence, although all reliability estimates in SAH and sepsis were generally inferior to those obtained in in healthy volunteers, all reliability estimates were surprisingly poor across all TFA metrics in all three groups.

Temporally separating the 5 min recordings had varying effects on the different TFA metrics; the SRD for gain more than doubled in healthy volunteers, and a similar effect was observed on gain and normalized gain in SAH, and on phase in sepsis. These findings raise concerns about the substantial variability in TFA metrics depending on the timing of the recording in a given individual. This is likely to reflect non‐linearity and/or non‐stationarity between the ABP and MCAv signal, in addition to a relatively low signal‐to‐noise ratio (Giller & Mueller, 2003). The lack of any effect of separating the recording segments on ICC adds very little to the above in relationship to the relative reliability of the estimates, because ICC is sensitive to variations both within and between groups. Essentially, this implies that if ICC is reported on a highly heterogeneous population, a high within‐group SD could result in a high ICC, regardless of the flaws of the method (Hartmann et al., 2023).

In theory, one way of overcoming non‐linearity, non‐stationarity and/or a relatively low signal‐to‐noise ratio between ABP and MCAv is to extend the recoding duration. In alignment with current recommendations (Panerai et al., 2023), this did not markedly affect the reliability of TFA metrics in healthy volunteers in the present study. Nevertheless, by using the expanding window sensitivity method (Mahdi et al., 2017; Schönbrodt & Perugini, 2013), we found that a minimum recording duration of 8 min was necessary if both stable gain and normalized gain measures were to be achieved, which stresses that further studies are required to determine the optimal recording duration in healthy individuals.

The effect of extending the recording duration on test–retest reliability differed between the two patient groups. In SAH, the SRD for normalized gain was markedly reduced, with a similar trend for gain; in sepsis, the only effect was a slight increase in CV for coherence. In any event, the expanding window sensitivity showed that the necessary recording durations for achieving stable TFA metrics in SAH and sepsis were 12 and 14.5 min, respectively. These findings are consistent with the notion that prolonging the recording duration might reduce susceptibility to non‐stationarity, non‐linearity and noise, especially in patient populations (Giller & Mueller, 2003). Nonetheless, it must be noted that expanding the recording duration from 5 to 12 min in patients with SAH did not markedly affect the reliability of these metrics. Furthermore, the required recording times varied markedly for phase, and, combined with the surprising finding that it could not be defined at all in healthy individuals, this might call for a critical reassessment of its use as a measure of dCA, at least when based on spontaneous ABP oscillations. In contrast, coherence generally provided similar absolute and relative reliability estimates regardless of recording duration and temporal separation, suggesting that this is a relatively stable measure of the degree of linearity between ABP and MCAv, although notably different recording duration of ≤13.5 min might be required.

Rather than extending the recording duration, a more effective means of overcoming the impact of non‐linearity, non‐stationarity and/or a relatively low signal‐to‐noise ratio between ABP and MCAv on TFA is to increase ABP variability experimentally. Several protocols have been established to achieve this, of which repeated squat–stands are the most thoroughly studied that have been reported to provide superior within‐ and between‐day CV values when compared with TFA based on spontaneous ABP oscillations (Burma et al., 2020; Smirl et al., 2015). However, in conditions such as SAH and sepsis, where patients might be unconscious or have mobility impairments, these physical manoeuvres are often not feasible, mainly owing to patient safety concerns. Consequently, the effectiveness of alternative techniques to induce input power, such as oscillatory lower‐body negative pressure or cyclic thigh‐cuff inflation–deflation, on the test–retest reliability remains to be evaluated in future studies.

Several limitations of this study deserve mention. First and foremost, this is a secondary analysis from previous studies with different purposes and designs (Berg et al., 2015, 2012; Berg, Plovsing, et al., 2013; Berg & Plovsing, 2016; Olsen et al., 2022), and although both ABP and MCAv were recorded in the same way, and TFA was conducted using identical methods, unintentional confounders might be present. These unintentional confounders include the possible differences of results from the recommended settings in the CARNet white paper (Panerai et al., 2023), and the default settings in the scripts used in this manuscript (Elting et al., 2020). Furthermore, the absolute TFA metrics might be affected by the medications administered in the two patient groups, but it should not influence the reliability estimates, because they were not changed throughout the whole recording period. As another limitation, the variation in recording duration and the duration of the break between temporally separated recordings might have affected some of the reliability measures, in addition to the lack of internal consistency for coherence in SAH when comparing consecutive 5 min recordings and phase. In healthy volunteers when comparing the first half and last half of the recordings, the specific reliability measures relating to these comparisons must be interpreted with caution. Furthermore, we had no data to assess the impact on reliability of temporally separating recordings longer than 10 min, and we were unable to assess the impact of using recordings in accordance with the point of stability in groups other than SAH. We also did not continually monitor end‐tidal or transcutaneous $P_{CO_{2}}$ in our patients, which would have been favourable here, because changes in arterial carbon dioxide tension during the recording period might profoundly affect MCAv independently of ABP, and thus contribute to non‐stationarity and non‐linearity (Ogoh et al., 2020). Although neither end‐tidal nor transcutaneous $P_{CO_{2}}$ was systematically monitored, this and other confounders were sought to be minimized in healthy subjects by having them positioned and resting for 20 min before recording; in 10 of the healthy volunteers in whom end‐tidal $P_{CO_{2}}$ was monitored, this remained stable in all cases (Berg, Plovsing, et al., 2013). For the two patient groups, which were both mechanically ventilated, the tidal volume, respiratory rate and the infusion rates of vasopressors and sedatives were all kept constant immediately before and during the recordings. However, further studies in which end‐tidal $P_{CO_{2}}$ is continually monitored are required to verify the absolute and relative reliability estimates reported here. Furthermore, in terms of the patient group, we included only patients with SAH and sepsis; our findings in non‐healthy individuals thus apply only specifically to these two patient groups.

In conclusion, a recording duration of 5 min appears to be insufficient for obtaining stable and reliable TFA metrics when based on spontaneous ABP oscillations, particularly in critically ill patients with SAH and sepsis. However, this must be verified in future studies, in which end‐tidal $P_{CO_{2}}$ is continually monitored in accordance with the current CARNet recommendations. Furthermore, additional studies are required to identify the optimal recording durations for all TFA metrics, in terms of both stability and test–retest reliability, and to establish whether experimental protocols that force ABP oscillations might be used favourably in these patient populations.

## AUTHOR CONTRIBUTIONS

Markus Harboe Olsen, Christian Riberholt, Kirsten Møller and Ronan M. G. Berg designed the study; Markus Harboe Olsen, Christian Riberholt, Ronni R. Plovsing and Ronan M. G. Berg collected the data; Markus Harboe Olsen did the analyses; all authors interpreted the data; Markus Harboe Olsen and Ronan M. G. Berg wrote the first draft; all authors revised and approved the final version of the manuscript and agree to be accountable for all aspects of the work in ensuring that questions related to the accuracy or integrity of any part of the work are appropriately investigated and resolved. All persons designated as authors qualify for authorship, and all those who qualify for authorship are listed.

## CONFLICT OF INTEREST

The authors have no conflict of interest to report.

## Data Availability

The data underlying our findings can be shared upon reasonable request directed to the corresponding author.
